# The effect of a collar on primary stability of standard and undersized cementless hip stems: a biomechanical study

**DOI:** 10.1007/s00402-024-05374-7

**Published:** 2024-05-18

**Authors:** Manuel Kistler, Arnd Steinbrück, Florian Schmidutz, Alexander C. Paulus, Boris Michael Holzapfel, Matthias Woiczinski

**Affiliations:** 1 Department of Orthopaedics and Trauma Surgery, Musculoskeletal University Center Munich (MUM), University Hospital, LMU, Marchioninistraße 15, 81377 Munich, Germany; 2 Orthopaedic Surgical Competence Center Augsburg (OCKA), Vinzenz-Von-Paul-Platz 1, 86152 Augsburg, Germany; 3 Orthopaedic Center Rosenheim, Äußere Münchener Straße 94, 83026 Rosenheim, Germany; 4 Orthopaedic Specialist Center Weilheim, Johann-Baur-Str. 5, 82362 Weilheim, Germany; 5 Experimental Orthopaedics University Hospital Jena, Campus Eisenberg, Waldkliniken Eisenberg, Eisenberg, Germany

**Keywords:** Biomechanics, Total hip arthroplasty (THA), Primary stability, Micromotion, Collar, Undersizing

## Abstract

**Introduction:**

Aseptic loosening and periprosthetic fractures are main reasons for revision after THA. Quite different from most other stem systems, Corail cementless hip stems show better survival rates than their cemented counterpart, which can possibly be explained by the use of a collar. The study aimed to investigate primary stability with standard and undersized hip stems both collared and collarless.

**Materials and methods:**

Primary stability of cementless, collared and collarless, femoral stems was measured in artificial bones using both undersized and standard size. After preconditioning, 3D micromotion was measured under cyclic loading at the bone-implant interface.

**Results:**

The use of a collar resulted in higher micromotion within the same stem size but showed no statistically significant difference for both standard and undersized hip stems. The collared and collarless undersized stems showed no significant differences in 3D micromotion at the upper measuring positions compared to the standard stem size. Micromotion was significantly higher in the distal measuring positions, with and without collar, for the undersized stems (vs. standard collarless stem size).

**Conclusion:**

The key finding is that the collarless and collared Corail hip stems, within one stem size, showed no significant differences in primary stability. Undersized stems showed significantly higher micromotion in the distal area both with and without collar.

## Introduction

The number of primary total hip arthroplasties (THA) is increasing annually. Especially aseptic loosening (22.7%) and periprosthetic fractures (15.9%) are major reasons for revision [1]. Even though cemented THAs can significantly reduce periprosthetic fractures, the usage of primary cementless stems is increasing [1, 2]. Possible risk factors for periprosthetic fractures are age, sex, the use of cementless stems, and movement of the implant (micromotion or subsidence) [2–4]. For cementless stems, in particular, many publications refer to an early in vivo research study with an osseointegration limit of 150 µm of implant movement (micromotion) [5]. A recent systematic review showed ranges between 15 µm and 750 µm for osseointegration [6]. Nevertheless, periprosthetic fractures and aseptic loosening are associated with micromotions [7].

As shown in the National Joint Registry (NJR), cemented stems tend to have better survival rates than cementless stems [8]. This is mainly due to the better performance of cemented stems in the older patient population. Interestingly, the cementless Corail, however, demonstrated better survival rates than its cemented counterpart. This might be due to its collared stem design which seems to reduce the risk for periprosthetic fractures [2]. In particular, cementless collared hip stems have been shown to be associated with significantly lower failure rate after approximately 17 years (collared 1.4%, collarless 2.9%) in the NJR [9] as well as in the German Arthroplasty Registry (EPRD) [2, 10, 11]. Demey et al. showed in a static test significantly higher push-out stability until fracture or subsidence in the vertical and horizontal planes for cementless collared femoral stems, which might be a reason for the enhanced survival rates [12].

Preoperative templating does not always reliably predict definitive stem size. Despite the greater amount of bone removal, oversized hip stems provide better primary stability and overall stiffness [13]. However, they also significantly increase cortical stress, which may be associated with a higher risk of periprosthetic fractures [14]. To avoid the incidence of intraoperative femoral fractures resulting from the use of an oversized stem, surgeons sometimes choose a smaller (undersized) stem than planned. Whether intentionally or unintentionally, undersized femoral stems reveal significantly higher micromotions [15]. Compared to the standard size, especially bones with undersized Corail stem create more radiolucent lines in cementless hip arthroplasty which, therefore, possibly reduces the lifetime of the implant [16, 17]. Hiskins et al. reported a four times higher revision rate with undersized Corail stems [18]. The impact of the collar, however, was not investigated in their study. There is currently the hope that cementless undersized hip stems with collar will achieve sufficient primary stability compared to the collarless stem design with the same size.

The effect of a collar on micromotions of an undersized collared hip stem has not been studied experimentally so far. Thus, the aim of this study was to investigate in vitro micromotions in artificial bones of both collared and collarless, standard and undersized, hip stems. We hypothesized that in case of undersizing, collared hip stems would achieve sufficient primary stability compared to collarless stems, both in the control group as well as in the undersized group.

## Materials and methods

### Implant and specimen preparation

**Fig. 1:**
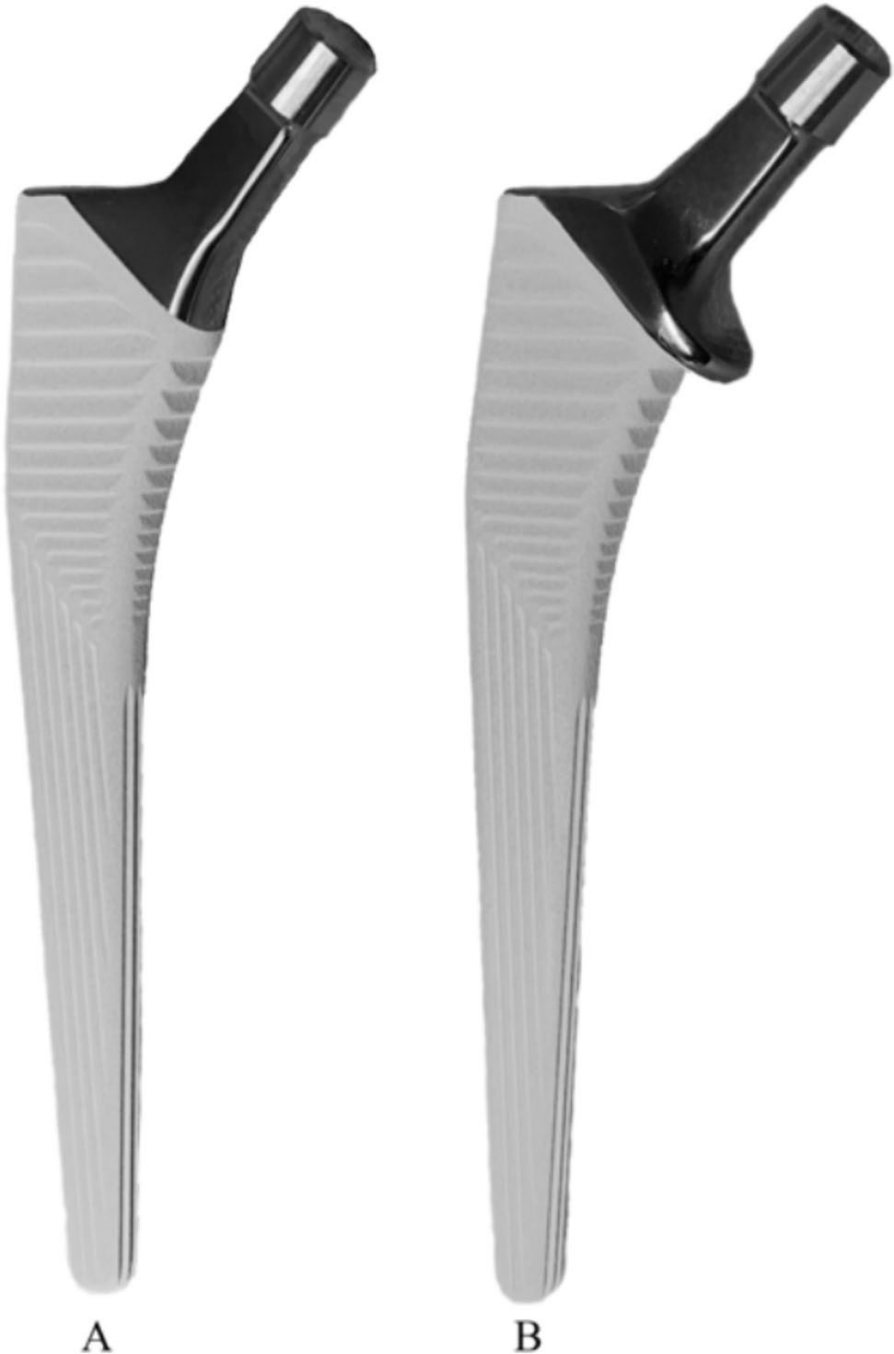
Collarless (**A**) and collared (**B**) HA coated femoral stem both with 135° neck angle

All experiments were performed with the cementless HA (hydroxyapatite) coated version of the CORAIL^®^ femoral stem (DePuy Synthes, Raynham, MA, U.S.) with and without collar, having a neck angle of 135° and a medium length ceramic head with a diameter of 32 mm (12/14 taper) (Fig. 1). Based on digital preoperative X-ray templating (mediCAD 2D, version 6.5, mediCAD Hectec GmbH, Altdorf/Landshut, Germany) of the artificial bones, sizes 15 and 13 were determined for the control group and undersized group, respectively. A total of 28 stems, divided into four groups of seven stems each (n = 7), were implanted in large left composite femurs (#3406, 4th generation, 17 PCF Solid Foam Cancellous, Sawbones® Pacific Research Laboratories, Vashon Island, WA, U.S.). An experienced senior orthopaedic surgeon, familiar with the stem system, performed the standardized implantation according to the manufacturer’s recommendations using rasps increasing in size until the final size was reached. The final rasp size was set equal to the final stem size resulting in a proximal press-fit fixation due to the compression rasp and the stem design. For this, an automatic impact device with constant energy was used (KINCISE™, DePuy Synthes, Raynham, MA, U.S.). After the stem insertion, radiographs were taken in two planes to ensure the correct stem position. Before distal femoral resection at 230 mm below the lesser trochanter, the anatomical epicondyle axis was transferred to the femoral midshaft to achieve standardized alignment of a neutral ante/retroversion [19]. Afterwards, all femurs were embedded in metal pots (80 mm high) using a polyurethane resin (RenCast® FC 52/53 Isocyanate & FC 53 Polyol, Huntsman Advanced Materials (Europe) BV, Everberg, Belgium). According to in vivo measurements of the hip, the specimens were aligned with an adduction angle of 16° in the frontal plane and a flexion angle of 9° in the sagittal plane to ensure the correct in vivo load direction [20–22].

## Measuring primary stability (Micromotion)

**Fig. 2:**
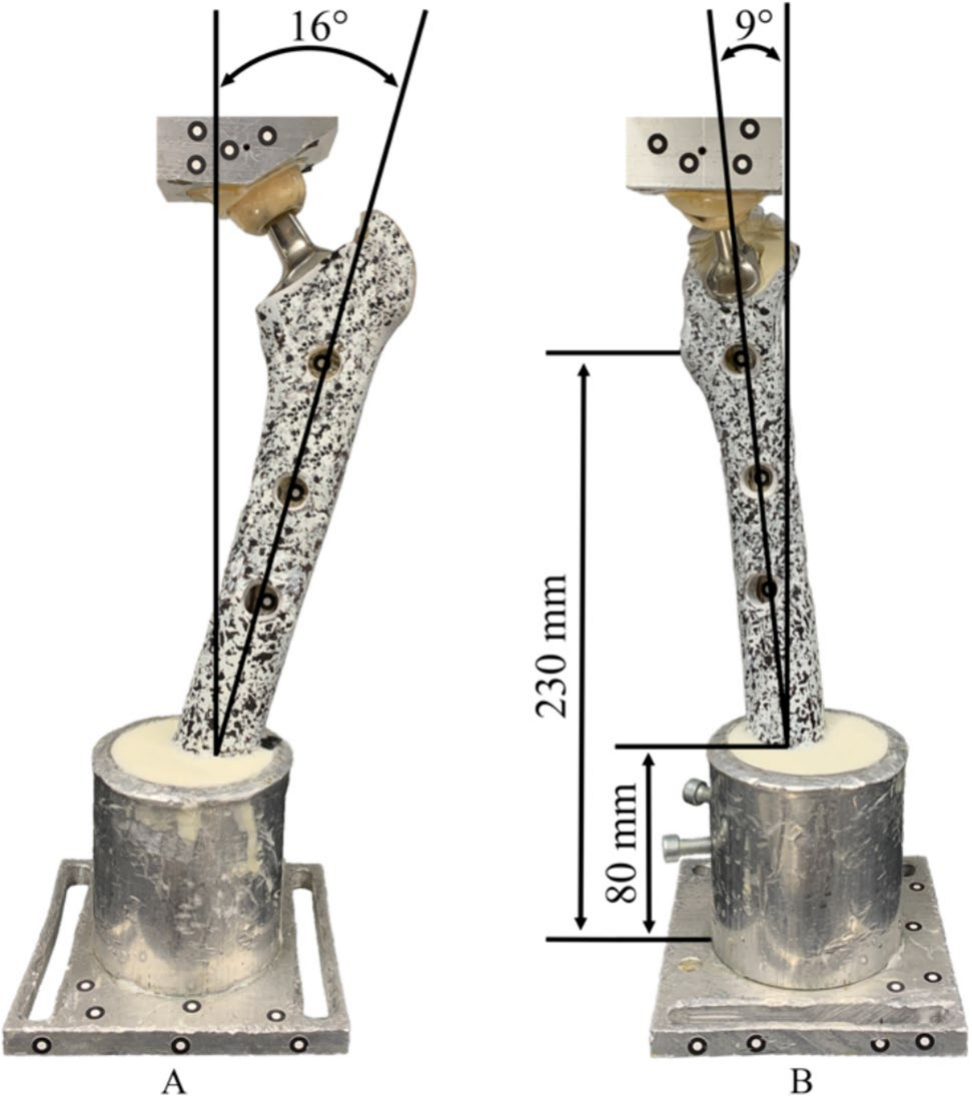
Ventral (**A**) and medial (**B**) artificial bone configuration, with applied stochastic pattern, embedded in a metal pot to ensure the connection to the universal material testing machine. Load was applied via a ceramic-on-ceramic joint. Optically visible measuring positions (proximal/midpoint/distal) for detection of stem movement

The degree of primary stability was defined as the magnitude of micromotion at the bone-implant interface. Based on three-dimensional (3D) digital image correlation, 3D micromotions were registered for every single point at six measuring positions: three on the medial and three on the ventral side (Fig. 2). The proximal points were located at the level of the lesser trochanter and the distal points 15 mm above the tip of the stem. The midpoints were centered between the proximal and distal points. An optical system consisting of two cameras with a resolution of 1936 × 1216 pixels (ARAMIS 3D Camera 2.3 M, Carl Zeiss GOM Metrology GmbH, Braunschweig, Germany), with a measuring distance of around 700 mm and a measuring volume of 560 mm × 380 mm × 380 mm was used for all measurements. The approximate measuring accuracy was 11.2 µm in focus-plane and 22.4 µm out of focus-plane, and the accuracy of strain measurements was approximately 0.1%. To allow detection of the prosthesis movement by the optical system, holes (10 mm diameter) were drilled into the bone at the mentioned measuring positions, and marker points were glued directly onto the stem. Next, a stochastic pattern was applied to the bone by spraying a thin coat of white paint and a speckle pattern of black paint (Aqua Eco + , European Aerosols GmbH, Hassmersheim Germany). After preconditioning, data was acquired at 5 Hz.

## Loading protocol

All specimen were loaded with the same sinusoid dynamic loading profile to determine the degree of primary stability. According to in vivo measurements [20, 23], all specimens were tested dynamically between 300 and 1700 N at a frequency of 1 Hz generated by a universal material testing machine (ElectroPuls E10000, Instron, Norwood, MA, U.S.). This simulated a person with approximately 70 kg during level walking. For preconditioning this loading pattern was applied for 600 cycles to avoid subsidence of the prosthesis during the measurement. Subsequently, each measurement position was recorded simultaneously and evaluated for 100 additional cycles. To avoid shear forces, the load was applied via a ceramic-on-ceramic joint (32 mm diameter), which was fixed to a X–Y linear guideway. This physiological loading profile has been used in previous studies.

## Data analysis and statistics

Data from all 28 specimens were analyzed using MATLAB (Version R2022a, MathWorks Inc., Natick, MA, USA). Relative 3D micromotion calculation was performed by subtracting the movement between bone and hip stem for each of the six measuring positions at every single cycle. The bone motion from four surrounding measurement points of the stochastic pattern at the relevant test positions was averaged. This calculation was done in each coordinate axis (x, y and z) and afterwards converted into 3D micromotion. The resulting micromotion and standard deviation (SD) were calculated from 100 cycles for each measuring position.

Statistical analysis was performed in 9. GraphPad Prism 9 (Version 9.5.1, GraphPad Software, Inc., La Jolla, USA). The calculated data of all specimens at all measuring positions were analyzed for normal distribution using the Shapiro–Wilk test and Kolmogorov–Smirnov test and checked for variance homogeneity using Brown-Forsythe test and Bartlett's test. One-way ANOVA was performed to compare the normal distributed micromotion data of standard and undersized collared and collarless hip stems. Tukey test was used for multiple comparison. P-values of < 0.05 were considered significant.

## Results

**Fig. 3:**
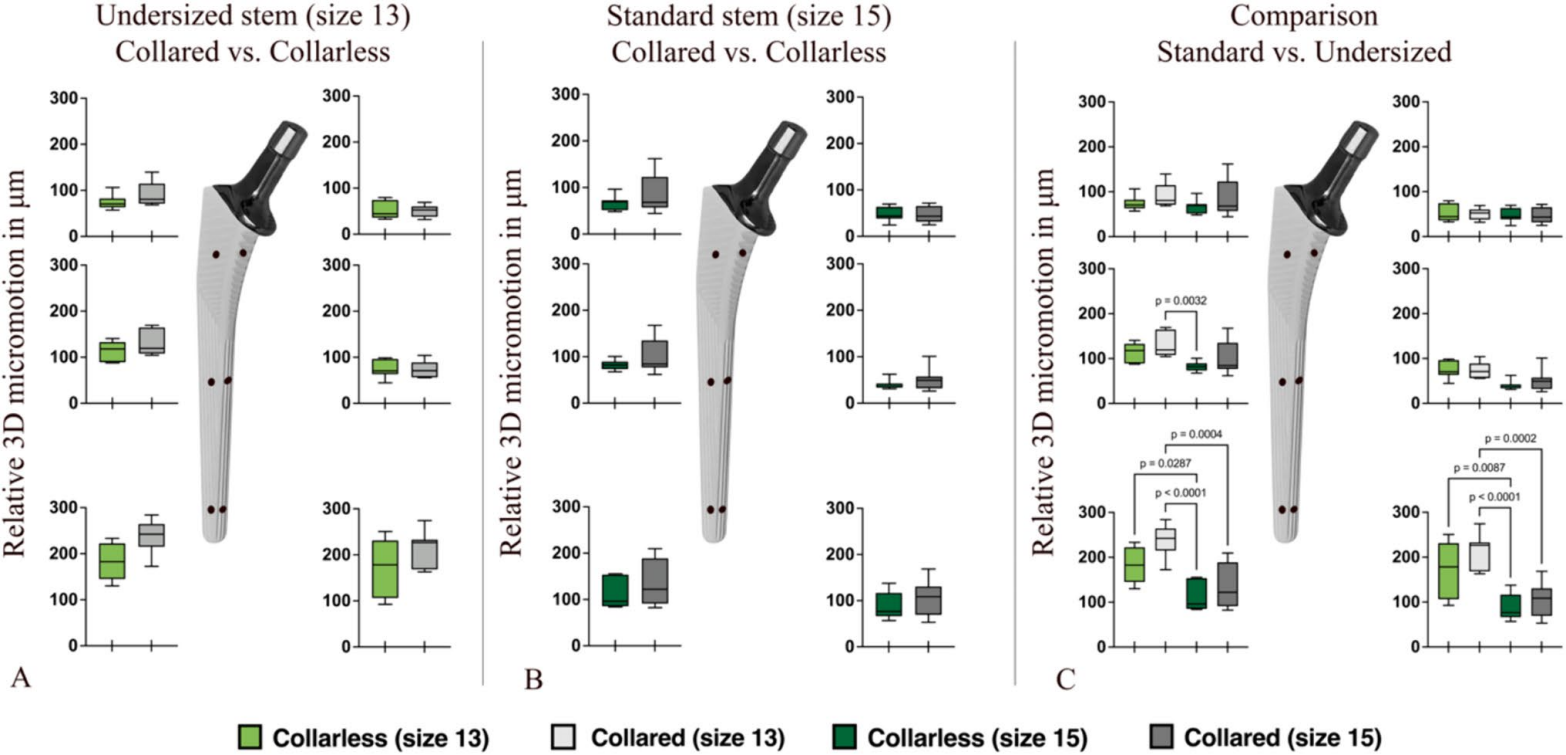
3D relative micromotion of collared and collarless hip stems at the six measuring positions for (**A**) undersized stem size 13 and (**B**) standard stem size 15. Micromotion of undersized collared and collarless stems in comparison to standard collared and collarless stem sizes (**C**). P-values indicate significant differences

All micromotion data followed a normal distribution and indicated homogeneous group variance. Micromotion using a collar showed no significant differences in both standard and undersized hip stems. Depending on the measuring position, the micromotion values increased from proximal to distal (Fig. 3A, B).

The 3D micromotion observed at the undersized hip stem ranged between 50.6 µm ± 12.6 µm and 237.7 µm ± 36.7 µm in comparison to the standard hip stem size (from 39.7 µm ± 10.7 µm to 132.0 µm ± 48.8 µm) (Table 1). Statistical analysis showed no significant differences at the upper measuring positions (proximal and medial midpoint) when using a collared and collarless undersized stem. Only the ventral midpoint showed significantly higher micromotions when using collared undersized hip stems compared to a standard stem size without collar. At the distal position, undersized stems showed significantly higher micromotions, for both collared and collarless vs. the collarless standard stem size (Fig. 3C).

**Table 1:** Values (in µm) of the primary stability (micromotion) at different measuring positions depending on the stem size and the use of a collar

Micromotion in µm								
	Undersized hip stem (size 13)							
	No Collar				Collar			
	Ventral		Medial		Ventral		Medial	
	Mean	SD	Mean	SD	Mean	SD	Mean	SD
Proximal	74.7	16.6	51.4	18.8	90.5	26.7	50.6	12.7
Midpoint	114.8	20.1	74.7	19.4	133.9	29.8	73.5	18.2
Distal	183.3	38.0	172.4	58.4	237.7	36.7	217.0	39.0

	Standard hip stem (size 15)							
	No Collar				Collar			
	Ventral		Medial		Ventral		Medial	
	Mean	SD	Mean	SD	Mean	SD	Mean	SD
Proximal	68.4	15.9	47.4	15.7	91.6	43.3	48.4	17.7
Midpoint	83.0	11.0	39.7	10.7	101.3	37.3	51.5	24.3
Distal	109.0	31.8	88.8	28.9	132.0	48.8	105.5	39.2

## Discussion

The most important finding of this study is that the collarless and collared Corail hip stems, within the same stem size, showed no significant differences in primary stability. Undersized stems showed significantly higher micromotion at the ventral midpoint and in the distal area when compared to standard size stems but only exceeded the osseointegration limit of 150 µm by a maximum of 88 µm.

Using a collared hip stem, our study observed slightly higher micromotion compared to the use of collarless stems. This result is, however, not statistically significant. Due to the proximal anchoring stem design, the distal micromotion increased. This effect was also seen in other THAs and prevents distal stiffening of the bone as well as stress shielding. A finite element model by Kadir et al. also could not demonstrate an effective reduction in micromotion using collared hip stems [24]. This could be due to the fact that the collar prevents subsidence, which reduces further press-fit and therefore decreases primary stability of the stem. This in turn creates a stirring movement of the distal stem tip. Using artificial bones could prevent this subsidence as these are known to have higher density of cancellous and cortical bone than human bone. Therefore, even holes in the artificial bones barely influence bone mechanics. A cadaver study by the research group of Camine et al. also showed higher micromotions and reduced subsidence in human specimens using collared hip stems [25]. On the other side, some studies have shown a reduction in the risk of periprosthetic fractures with the use of a collar [2, 26]. Collared femoral stems can also withstand more vertical and horizontal forces before subsidence or fractures occur [12], but less subsidence can lead to a reduced risk for periprosthetic fractures. A correlation between periprosthetic fractures and increased micromotions with the use of a collar has not yet been investigated.

In our study, the use of undersized femoral stems with and without collar, showed significantly higher micromotions at the ventral midpoint and in the distal area but femoral stems with a proximal fixation zone seem to achieve adequate primary stability. However, recent studies have shown that undersizing significantly reduces primary stability and increases rate of early subsidence and aseptic loosening [14–16]. Hoskins et al. also published data from the Australian Joint Replacement Registry showing significantly higher revision rates for undersized Corail stems [18].

Considering only the micromotion limits for osseointegration, the biomechanical findings of our study vary between 39.7 µm ± 10.7 µm and 237.7 µm ± 36.7 µm, depending on the measuring position (Table 1). The highest micromotions were detected in the distal area of the collared and collarless undersized stems. Only these distal values exceeded the limit of 150 µm that has been defined for good implant osseointegration in the literature [5]. A recent work by Kohli et al. showed different micromotion limits for successful implant osseointegration, reaching values up to 750 µm [6]. Therefore, type and design of the implant and external factors must be considered when evaluating micromotion for successful osseointegration. In the present study, micromotions of undersized stems exceeded the value of 150 µm by a maximum of 88 µm, both with and without collar, and therefore osteointegration may be possible.

The main limitation of this study is the use of artificial bones to investigate the primary stability of the Corail femoral stem. Clinically relevant anatomical deformities, such as coxa vara/coxa valga also have an influence on the lever arms acting in the hip joint. This has the effect of influencing the load application on the hip stem, which in turn affects the primary stability. Furthermore, a high activity level would also change this load condition. Even though the absolute findings cannot be transferred to human bones, composite bones allow a more standardized comparison within themselves and with other studies [15, 19, 27].

Second, this biomechanical model is a simplification without any muscle forces or tension band effect, such as the tractus iliotibialis. Other experimental set-ups of both human and artificial bones are also described in the literature without the use of muscle tensions [12, 15, 19, 24–27]. Stiffening the bone due to this tension band effect resulted in reduced bone bending, which can lead to decreased micromotions. Thus, this configuration simulated extreme conditions, which can lead to increased micromotions. Furthermore, the used physical load application simulated only level ground walking and not extreme loads as stumbling.

Overall, our study was able to show no significant differences in micromotion within the same stem size using a collared and collarless Corail femoral stem. However, the results show a tendency to increased micromotions when using a collar. Undersized stems showed significantly higher micromotions in the distal area. Further studies using fresh frozen cadaveric specimens are required for clinical validation of these results, in particular when the influence of human anatomical variations should be investigated.

## Conclusion

According to our in vitro study using composite femora, there is a tendency of more micromotion in the collared compared to the collarless Corail stem though these differences did not reach statistical significance. Due to the reduced risk for periprosthetic fractures, however, the use of a collared stem has been recommended. From a clinical perspective, these results suggest that a surgeon who uses a collared stem might mitigate the risk of periprosthetic fractures but might at the same time risk higher micromotions.

Undersizing both designs showed significantly higher micromotions in the distal area but only exceed the osseointegration limit by a maximum of 88 µm. We therefore advise surgeons to avoid undersizing even in collared stems due to the higher rate of revision.

## Ethical approval

This is a biomechanical study without human biological material. The Ludwig-Maximilians-University Munich Research Ethics Committee has confirmed that no ethical approval is required.

## Data Availability

The datasets generated and analyzed during the current study are available from the corresponding author (Manuel Kistler) on request.
